# Type and approach of hysterectomy and oncological survival of women with stage II cancer of endometrium: a large retrospective cohort study

**DOI:** 10.3389/fonc.2024.1404831

**Published:** 2024-05-13

**Authors:** Xu Shuai, Dan Xiao, Binhua Han, Yixue Du

**Affiliations:** ^1^ Department of Obstetrics and Gynecology, Chengdu Fifth People’s Hospital (The Second Clinical Medical College, Affiliated Fifth People's Hospital of Chengdu University of Traditional Chinese Medicine), Chengdu, China; ^2^ Geriatric Disease Institute of Chengdu, Cancer Prevention and Treatment institute of Chengdu, Chengdu, China; ^3^ Department of Obstetrics and Gynecology, West China Second University Hospital, Sichuan University, Chengdu, China

**Keywords:** endometrial cancer, hysterectomy, laparoscopy, laparotomy, disease-free survival, overall survival

## Abstract

**Objective:**

To explore the association between the Type and approach of hysterectomy and oncological survival of women with stage II cancer of the endometrium

**Patients and methods:**

684 women with stage II endometrial cancer were included. Eligible cases were grouped by type of hysterectomy (simple hysterectomy or radical hysterectomy)and approach of hysterectomy (laparoscopy or laparotomy). The baseline characteristics were compared among groups. The survival outcomes (disease-free survival and overall survival) were calculated and compared among groups, and the underlying confounding factors were adjusted by the Cox proportional hazard regression analysis.

**Results:**

The radical hysterectomy group and the simple hysterectomy group had 217 cases and 467 cases, respectively. Between the groups, the difference in 5-year disease-free survival (87.3% versus 87.9%, HR=0.97, *P*=0.87) and 5-year overall survival (83.8% versus 83.8%, HR=0.95, *P*=0.95) was not statistically significant. The laparotomy group and the laparoscopy group had 277 cases and 407 cases, respectively. Between the groups, the difference in 5-year disease-free survival (88.7% versus 87.1%, HR=1.22, *P*=0.34) and 5-year overall survival (85.5% versus 82.7%, HR=1.00, *P*=0.99) was not statistically significant.

**Conclusion:**

For long-term oncological survival, radical hysterectomy is not superior to total hysterectomy in stage II endometrial cancer. Also, for stage II cancer of the endometrium, laparoscopic hysterectomy is as oncologically safe as open hysterectomy.

## Introduction

With the increase in the elderly population and obesity population, endometrial cancer has become the most common malignancy of the female reproductive system in high-income countries (1–3). Although the age-standardized incidence of endometrial cancer is on the rise worldwide, in contrast, the mortality rates associated with endometrial cancer have decreased over the same period by 15 percent (4). This is largely due to progress in the diagnosis and treatment of endometrial cancer (1–3). However, endometrial cancer remains a serious threat to women’s health.

Generally, cancer of the endometrium is staged by the International Federation of Gynaecology and Obstetrics (FIGO) staging system for endometrial cancer (1, 2, 5, 6). The FIGO 2009 stage II endometrial cancer includes cases in which the tumor invades cervical stroma, the reported 5-year survival is 74% (1, 2, 5, 6). The standard surgical management for apparent early-stage cancer of the endometrium includes hysterectomy, bilateral salpingo-oophorectomy, and lymphadenectomy (1, 2, 5). Based on the findings of some prospective clinical researches, a minimally invasive approach is recommended for endometrial cancer by many societies (1–3, 5, 6). They concluded that minimally invasive surgery for endometrial cancer is as safe as traditional open surgery (1–3, 5–7). However, about five years ago, two large clinical trials confirmed that minimally invasive surgery is not oncologically safe for cervical cancer (8, 9). Since then, more and more studies have confirmed the same conclusion (10–13). So we have this question: is a minimally invasive approach safe for stage II cancer of the endometrium which is also cervical involvement? In addition, in the treatment of stage II cancer of the endometrium, whether radical hysterectomy is oncologically superior to simple hysterectomy has not been determined (14–17).

Taken together, conducted at two high-volume Chinese centers, the current study aimed to answer the following two questions: (1) is laparoscopic hysterectomy as oncologically safe as open hysterectomy for stage II cancer of the endometrium? (2) is radical hysterectomy oncologically superior to simple hysterectomy in stage II cancer of the endometrium?

## Patients and methods

### Study design

Conducted at two Chinese high-volume hospitals, our study was a retrospective cohort study. Considering the fact that this study did not contain any identifiable private data and given the retrospective nature of this study, the ethics committee of the participating hospitals waived the ethical review and approval following the institutional requirements and local legislation. Meanwhile, this study was conducted strictly following the Declaration of Helsinki (18).

### Study cohort

The following two centers were involved in this study: West China Second University Hospital and Chengdu Fifth People’s Hospital. Patients with endometrial cancer who were managed in these centers between January 1, 2011 and December 31, 2018 were screened for eligibility for this study.

Cases were included in this study if they: (1) were under 70 years of age, (2) had histologically proven endometrioid adenocarcinoma, (3) were staged as FIGO 2009 stage II, (4) at least underwent a hysterectomy, bilateral salpingectomy, bilateral oophorectomy, and pelvic lymphadenectomy, and (5) were consecutively followed up at the participating centers.

Cases were not included in this study if they: (1) were not primarily managed by surgery, (2) underwent preoperative adjuvant chemotherapy or radiotherapy, (3) had a history of other cancer, (4) had a physical status score (by American Society of Anesthesiologists, ASA) of larger than III, (5) did not undergo systematic lymphadenectomy, or (6) were lost to postoperative follow-up.

### Data collection

Data of interest were collected using pre-designed spreadsheets, as follows: the date of diagnosis, patient’s age at diagnosis of endometrial cancer, patient’s marital status when they were diagnosed with endometrial cancer, patient’s body mass index (BMI) at diagnosis, the physical status score by ASA scoring system when they underwent surgical staging, the grade of tumor differentiation, the status of lymphovascular space invasion (LVSI), the size (diameter) of the primary tumor, the result of cytology of peritoneal flush fluid, the type of hysterectomy (simple or radical), the approach of surgery (laparotomy or laparoscopy), the type of systematic lymphadenectomy (pelvic plus para-aortic lymphadenectomy or pelvic lymphadenectomy), and postoperative adjuvant management (none, radiotherapy, chemotherapy, or chemoradiotherapy). In our study, a simple hysterectomy refers to the type A hysterectomy in the Querleu-Morrow classification, and a radical hysterectomy refers to the type C hysterectomy in the Querleu-Morrow classification (19).

Data on survival outcomes of interest were collected as follows: whether the patient is alive or not, the site of disease recurrence, the date of disease recurrence, the date and the cause of death. In the current study, all eligible cases were followed up until December 1, 2023 or death.

### Outcomes of interest

In this study, the primary outcomes of interest were 5-year disease-free survival (DFS) and 5-year overall survival (OS). DFS was defined as the interval between the date of surgery for endometrial cancer and the date of documented recurrence of endometrial cancer or death caused by endometrial cancer. OS was defined as the interval from the date of surgery for endometrial cancer to the date of documented death from any cause.

### Statistical analysis

Based on the type of hysterectomy, the eligible cases were divided into the simple hysterectomy group and the radical hysterectomy group. Also, based on the approach of surgical staging, the included cases were divided into the laparotomy group and the laparoscopy group. Standard descriptive statistics were employed to report the data regarding the characteristics of the study cohort (20, 21). Among the groups, the categorical variables were compared using the chi-squared test or the Fisher exact test, the continuous variables were compared using the *t*-test or the Wilcoxon rank-sum test (20, 21). The Kaplan-Meier method and the log-rank test were employed to estimate and compare the 5-year OS and the 5-year DFS among groups (22–24). Hazard ratio (HR) and 95% confidence interval (CI) were calculated (22–24). To control the confounding factors, we utilized the Cox proportional hazard regression model (25). Candidate variables that were presumed clinically significant or that had a *P* value of less than 0.20 on univariate analysis were included in the Cox proportional hazard regression model (8, 9).

In this study, A two-sided *P* < 0.05 was considered statistically significant. In our study, we conducted all statistical analyses by employing the IBM SPSS version 25 (SPSS Inc., Chicago, IL, USA), and we generated the Kaplan-Meier curves by Stata version 17 (Stata Corp., College Station, TX, USA).

## Results

### Characteristics of the study cohort

**Figure 1:**
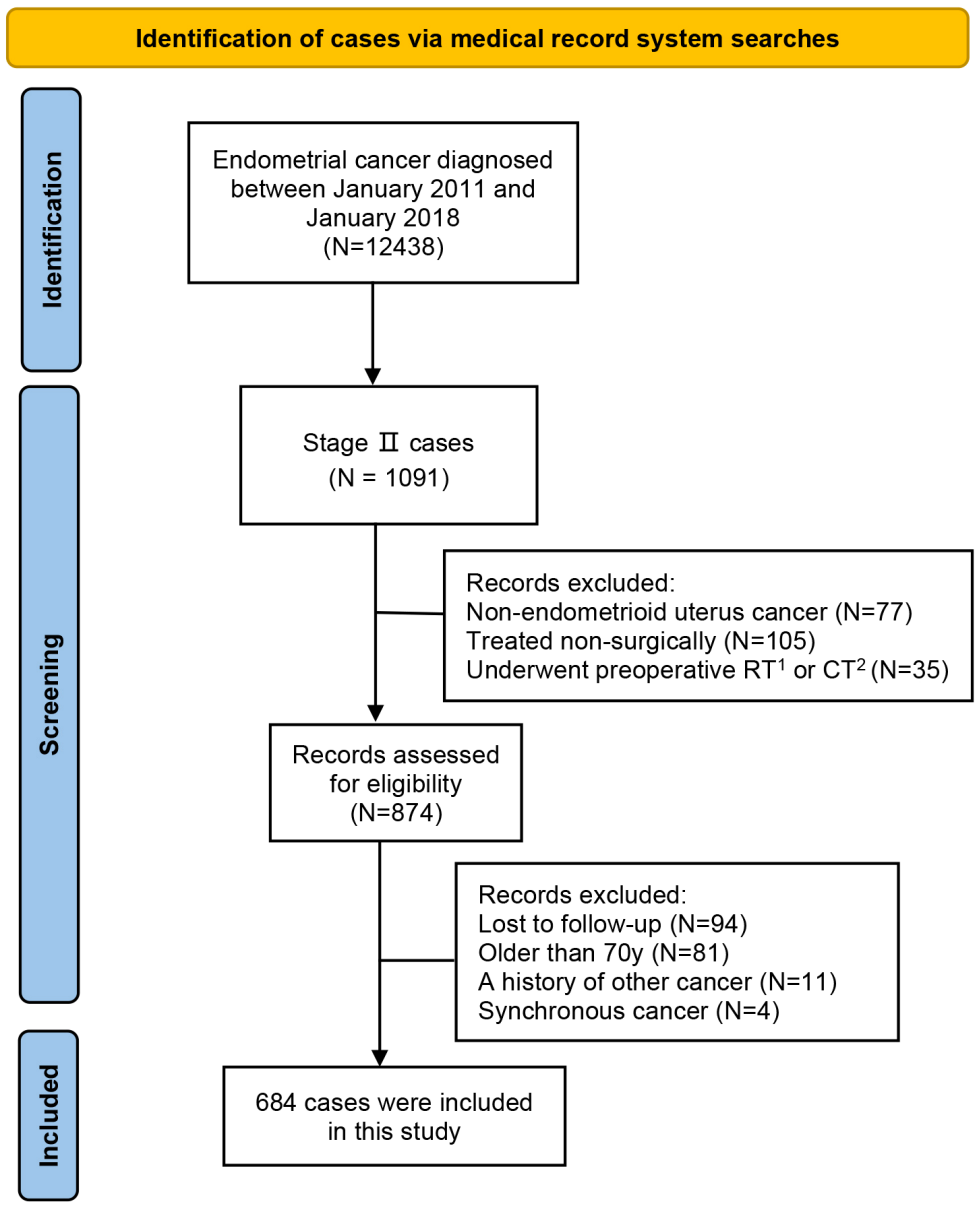
The case screening process.

Based on inclusion and exclusion criteria, 684 cases were eventually included in the study. **Figure 1** illustrates the case screening process. Among them, 277 patients got surgical management by open approach (the laparotomy group) and the remaining 407 cases underwent surgical staging by minimally invasive approach (the laparoscopy group). Based on the type of hysterectomy, a total of 217 cases were included in the radical hysterectomy group and 467 cases were included in the simple hysterectomy.

The mean age at diagnosis of the entire cohort was 57.9 years (standard deviation: 7.62), and the median follow-up duration was 77.0 months (range: 0–142). Of the included cases, 309 (45.2%) patients were older than 60 years. Most of the eligible cases had well-differentiated tumors, the proportion was 78.5% (537). 13.0% (89) of the included cases and 18.3% (125) of the included cases were identified with LVSI and positive peritoneal cytology, respectively. As for baseline data of treatment, 42.7% (292) of the included cases underwent combined pelvic and para-aortic lymphadenectomy, while approximately 13% of the eligible cases were not managed by any postoperative adjuvant therapy.

In terms of the baseline characteristics, there was no significant difference between the simple hysterectomy group and the radical hysterectomy group (or the laparotomy group and the laparoscopy group). However, a significant difference in age at diagnosis existed between the simple hysterectomy group and the radical hysterectomy group (*P*< 0.001). Also, the protocol of postoperative adjuvant therapy was significantly different between the laparoscopy group and the laparotomy group (*P*< 0.001). The baseline data of the study cohort is presented in **Table 1**.

**Table 1:** Characteristics of the study cohort^a^.

	Overall (N=684)	Surgical approach			Type of hysterectomy		
		The laparoscopy group (N=407)	The laparotomy group (N=277)	*P*	The radical hysterectomy group (N=217)	The simple hysterectomy group (N=467)	*P*
Years of diagnosis				0.638			0.098
2011-2014	297(43.4%)	180(44.2%)	117(42.2%)		105(48.4%)	192(41.1%)	
2015-2018	387(56.6%)	227(55.8%)	160 (57.8%)		112(51.6%)	275(58.9%)	
Age at diagnosis	57.9 ± 7.62	57.5 ± 7.80	58.4 ± 7.33	0.097	56.1 ± 7.9	58.7 ± 7.35	< 0.001
Age at diagnosis				0.696			0.161
< 60y	375(54.8%)	226(55.5%)	149(53.8%)		128(59.0%)	247(52.9%)	
≥60y	309(45.2%)	181(44.5%)	128(46.2%)		89(41.0%)	220(47.1%)	
Duration of follow-up	77.0 (0, 142)	53.0 (4.00, 131)	49.0 (4.00, 127)	0.215	82.0 (0, 142)	73.0 (2, 142)	0.173
Marital status at diagnosis				0.481			0.459
Married	379(55.4%)	221(54.3%)	158(57.0%)		116(53.5%)	263(56.3%)	
Single^b^	305(44.6%)	186(45.7%)	119(43.0%)		101(46.5%)	204(43.7%)	
BMI^c^ at diagnosis	21.1 ± 4.03	21.3 ± 3.92	20.7 ± 5.19	0.357	21.5 ± 5.44	20.9 ± 4.84	0.442
ASA^d^ physical status score				0.107			0.213
I/II	465(68.0%)	289(71.0%)	176(63.5%)		157(72.4%)	308(70.0%)	
III	219(32.0%)	118(29.0%)	101(36.5%)		60(27.6%)	159(30.0%)	
Grade				0.219			0.921
I or II	537(78.5%)	313(76.9%)	224(80.9%)		172(79.3%)	365(78.2%)	
III or undifferentiated	147(21.5%)	94(23.1%)	53(19.1%)		45(20.7%)	102(21.8%)	
Tumor size				0.219			0.803
< 4 cm	407(59.5%)	224(55.0%)	183(66.1%)		128(59.0%)	279(59.7%)	
≥ 4 cm	277(40.5%)	183(45.0%)	94(33.9%)		89(41.0%)	188(40.3%)	
LVSI^e^				0.187			0.080
No	595(87.0%)	355(87.2%)	240((86.6%)		185(85.3%)	410(87.8%)	
Yes	89(13.0%)	52(12.8%)	37(13.4%)		32(14.7%)	57(12.2%)	
Peritoneal cytology				0.081			0.911
Negative	559(81.7%)	338(87.0%)	221(79.8%)		175(81.6%)	384(82.2%)	
Positive	125(18.3%)	69(17.0%)	56(20.2%)		42(19.4%)	83(17.8%)	
Lymphadenectomy				0.095			0.873
Pelvic	392(57.3%)	244(60.0%)	148(53.4%)		124(57.1%)	268(57.3%)	
Pelvic and para-aortic	292(42.7%)	163(40.0%)	129(46.6%)		93(42.9%)	199(42.6%)	
Adjuvant therapy				< 0.001			0.080
CT^f^ or RT^g^	373(54.5%)	94(23.1%)	120(43.3%)		121(55.8%)	252(54.0%)	
CT plus RT	220(32.2%)	253(62.2%)	126(45.4%)		60(27.6%)	160(34.3%)	
No	91(13.3%)	60(14.7%)	31(11.2%)		36(16.6%)	55(11.8%)	

^a^Values are presented as mean ± standard deviation, median (minimum–maximum), or as number (percentage).

^b^Including never married, widowed, divorced, or separated.

^c^Body Mass Index.

^d^American Society of Anesthesiologists.

^e^Lymphovascular Space Invasion.

^f^Chemotherapy.

^g^Radiotherapy.

### Rates and sites of cancer recurrence

Of the included cases, 98 (14.3%) patients were identified with disease recurrence. There were 63 (15.5%) disease recurrences identified in the laparoscopy group, the number was 35 (12.6%) in the laparotomy group, and the statistical difference was not identified between the two groups (*P*=0.319). When the included cases were grouped according to the type of hysterectomy, as for the rate of cancer recurrence, there was still no significant difference identified between the groups (*P >*0.999). The cases of disease recurrence for the radical hysterectomy group and the simple hysterectomy group were 31 (14.3%) and 67 (14.3%), respectively.

In terms of the sites of cancer recurrence, the approach of surgical staging and the scope of hysterectomy did not affect the patterns of disease recurrence. For FIGO 2009 stage II endometrial cancer, the most common sites of disease recurrence were as follows: the abdomen (3.2%), the pelvis (3.1%), the regional lymph nodes (2.3%), and the lung (2.2%). In the entire study cohort, approximately 1% of cases underwent a multi-site tumor recurrence. The rates and patterns of cancer recurrences in the study cohort are presented in **Table 2**.

**Table 2:** Rates and sites of cancer recurrences^a^.

	Overall (N=684)	Surgical approach			Type of hysterectomy		
		The laparoscopy group (N=407)	The laparotomy group (N=277)	*P*	The radical hysterectomy group (N=217)	The simple hysterectomy group (N=467)	*P*
Recurrence				0.319			> 0.999
Yes	98(14.3%)	63(15.5%)	35(12.6%)		31(14.3%)	67(14.3%)	
No	586(85.7%)	344(84.5%)	242(87.4%)		186(85.7%)	400(85.7%)	
Site of recurrence							
Vagina	11(1.6%)	7(1.7%)	4(1.4%)	> 0.999	4(1.8%)	7(1.5%)	0.750
Pelvis	21(3.1%)	12(2.9%)	9(3.2%)	0.825	6(2.8%)	15(3.2%)	> 0.999
Nodal	16(2.3%)	11(2.7%)	5(1.8%)	0.608	5(2.3%)	11(2.4%)	> 0.999
Abdomen	22(3.2%)	13(3.2%)	9(3.2%)	> 0.999	7(3.2%)	15(3.2%)	> 0.999
Bone	8(1.2%)	6(1.5%)	2(0.7%)	0.484	3(1.4%)	5(1.1%)	0.713
Lung	15(2.2%)	10(2.5%)	5(1.8%)	0.421	4(1.8%)	11(2.1%)	> 0.999
Multiple	5(0.7%)	4(1.0%)	1(0.4%)	0.653	2(0.9%)	3(0.6%)	0.655

^a^Values are presented as numbers (percentages).

### Survival outcomes

**Figure 2:**
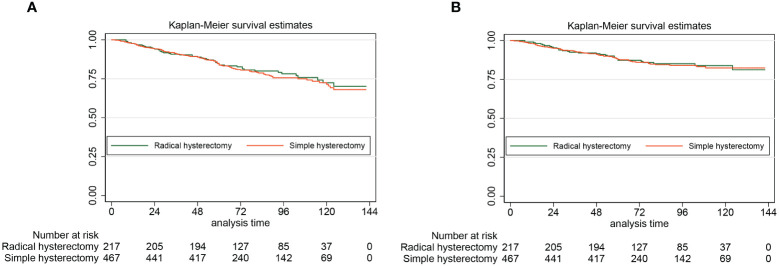
The Kaplan-Meier survival curves of the study cohort by radical hysterectomy versus simple hysterectomy (A for OS, B for DFS)

For women who got simple hysterectomy and women who got radical hysterectomy, the 5-year DFS rates estimated by the Kaplan-Meier method were 87.3% (95% CI: 81.9%-91.2%) and 87.9% (95% CI: 84.5%-90.6%), respectively. For patients with stage II cancer of the endometrium, undergoing radical hysterectomy did not improve the DFS when compared with undergoing simple hysterectomy (HR: 0.97, 95% CI: 0.63–1.84, *P*=0,87). For patients who underwent simple hysterectomy and patients who underwent radical hysterectomy, the 5-year OS rates estimated by the Kaplan-Meier method were 83.8% (95% CI: 80.0%-86.9%) and 83.8% (95% CI: 78.1%-88.2%), respectively. Similarly, for patients with stage II endometrial cancer, radical hysterectomy was not associated with a better 5-year OS (HR: 0.95, 95% CI: 0.67–1.33, *P*=0,95). **Figure 2** presents the Kaplan-Meier survival curves of the study cohort by radical hysterectomy versus simple hysterectomy (A for OS, B for DFS).

**Figure 3:**
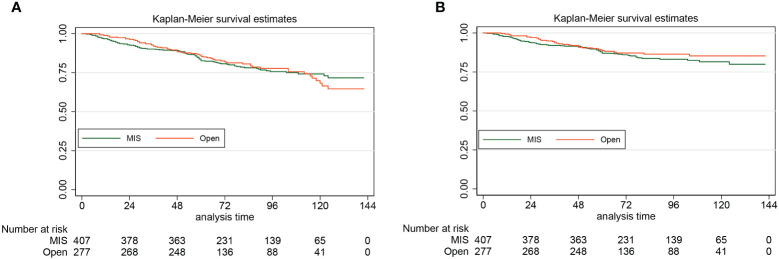
The Kaplan-Meier survival curves of the study cohort by laparotomy versus laparoscopy (A for OS, B for DFS)

For the laparoscopy group and the laparotomy group, the 5-year DFS were 87.1% (95% CI: 83.3%-90.0%) and 88.7% (95% CI: 84.2%-92.0%), respectively. By the Log-rank test, we found that for patients with stage II endometrial cancer, compared with the open approach, hysterectomy by minimally invasive surgery did not increase the risk of disease recurrence and cancer-specific death (HR: 1.22, 95% CI: 0.81–1.85, *P*=0.34). For the laparoscopy group, the 5-year OS was 82.7% (95% CI: 78.5%-86.1%). For the laparotomy group, the 5-year OS was 85.5% (95% CI: 80.7%-89.2%). Also, the approach of surgical staging did not affect the risk of all-cause death of women with stage II endometrial cancer (HR: 1.00, 95% CI: 0.73–1.39, *P*=0.99). The Kaplan-Meier survival curves of the study cohort by laparotomy versus laparoscopy (A for OS, B for DFS) are presented in **Figure 3**.

### Univariate analyses

For women with stage II endometrial cancer, by using the log-rank test, we found that the age at diagnosis (≥60 years versus<60 years: HR=2.45, 95% CI=1.76–3.41, *P*=0.00), the BMI at diagnosis (≥24 kg/m^2^ versus<24 kg/m^2^: HR=1.89, 95% CI=1.37–2.61, *P*=0.00), the grade of tumor differentiation (III/undifferentiated versus I/II: HR=2.32, 95% CI=1.66–3.23, *P*=0.00), and LVSI (positive versus negative: HR=12.49, 95% CI=8.63–18.08, *P*=0.00) were associated with the OS. While, the marital status at diagnosis (*P*=0.72), the ASA physical status score (*P*=0.53), the size of the primary tumor (*P*=0.13), the result of peritoneal cytology (*P*=0.11), the scope of lymphadenectomy (*P*=0.79), and the protocol of postoperative adjuvant therapy (chemotherapy/radiotherapy versus none: *P*=0.70, chemoradiotherapy versus none: *P*=0.26) did not affect the OS of women with stage II endometrial cancer.

For women with stage II cancer of the endometrium, the log-rank test shows that the age at diagnosis (≥60 years versus<60 years: HR=1.90, 95% CI=1.27–2.85, *P*=0.00), the BMI at diagnosis (≥24 kg/m^2^ versus<24 kg/m^2^: HR=1.75, 95% CI=1.17–2.60, *P*=0.01), the grade of tumor differentiation (III/undifferentiated versus I/II: HR=3.07, 95% CI=2.05–4.58, *P*=0.00), the size of primary tumor (≥4 cm versus<4 cm: HR=1.70, 95% CI=1.14–2.53, *P*=0.01), and LVSI (positive versus negative: HR=14.82, 95% CI=9.58–22.92, *P*=0.00) affected the risk of disease recurrence and death caused by cancer of the endometrium. While, the marital status at diagnosis (*P*=0.95), the ASA physical status score (*P*=0.77), the result of peritoneal cytology (*P*=0.09), the scope of lymphadenectomy (*P*=0.81), and the protocol of postoperative adjuvant therapy (chemotherapy/radiotherapy versus none: *P*=0.75, chemoradiotherapy versus none: *P*=0.90) did not have an effect on the DFS of patients with stage II cancer of the endometrium.

**Table 3** presents the results of univariate analyses.

**Table 3:** Univariate analyses of survival for stage II endometrial cancer.

	OS^a^			DFS^b^		
	HR^c^	95% CI^d^	*P*	HR	95% CI	*P*
Age at diagnosis						
< 60 years	1			1		
≥ 60 years	2.45	1.76-3.41	0.00	1.90	1.27-2.85	0.00
Marital status at diagnosis						
Married	1			1		
Single^e^	1.01	0.70-1.61	0.72	1.13	0.61-2.03	0.95
BMI^f^ at diagnosis						
< 24 kg/m^2^	1			1		
≥ 24 kg/m^2^	1.89	1.37-2.61	0.00	1.75	1.17-2.60	0.01
ASA^g^ physical status score						
I or II	1			1		
III	1.09	0.82-1.43	0.53	1.14	0.88-1.41	0.77
Grade						
I or II	1			1		
III or undifferentiated	2.32	1.66-3.23	0.00	3.07	2.05-4.58	0.00
Tumor size						
< 4 cm	1			1		
≥ 4 cm	1.28	0.93-1.77	0.13	1.70	1.14-2.53	0.01
LVSI^h^						
No	1			1		
Yes	12.49	8.63-18.08	0.00	14.82	9.58-22.92	0.00
Peritoneal cytology						
Negative	1			1		
Positive	1.75	0.89-2.14	0.11	1.81	0.91-2.23	0.09
Surgical approach						
Open	1			1		
MIS^i^	1.00	0.73-1.39	0.99	1.22	0,81-1.85	0.34
Type of hysterectomy						
Simple hysterectomy	1			1		
Radical hysterectomy	0.95	0.67-1.33	0.95	0.97	0.63-1.48	0.87
Lymphadenectomy						
Pelvic	1			1		
Pelvic plus para-aortic	1.32	0.87-1.83	0.79	1.34	0.85-1.77	0.81
Adjuvant therapy						
No	1			1		
CT^j^ or RT^k^	1.07	0.75-1.53	0.70	1.07	0.69-1.68	0.75
CT plus RT	0.71	0.39-1.29	0.26	1.04	0.54-2.00	0.90

^a^Overall survival.

^b^Disease-free survival.

^c^Hazard ratio.

^d^Interval confidence

^e^Including never married, widowed, divorced, or separated.

^f^Body mass index.

^g^American Society of Anesthesiologists.

^h^Lymphovascular space invasion.

^i^Minimally invasive surgery.

^j^Chemotherapy.

^k^Radiotherapy.

### Multivariate analyses

In the multivariate analyses, a Cox regression model was made for this study. We included factors that had potential clinical significance or that had a *P <* 0.20 on univariate analysis, as follows: BMI at diagnosis, age at diagnosis, the tumor differentiation grade, the size of the primary tumor, the result of peritoneal cytology, the status of LVSI, the approach of surgical staging, the type of hysterectomy, and the protocol of adjuvant therapy. According to the multivariate Cox regression analyses, for women with stage II endometrial cancer, we found that the approach of surgical staging and the type of hysterectomy did not affect the long-term DFS (laparoscopy versus laparotomy: aHR=1.04, 95% CI=0.68–1.60, *P*=0.85. radical hysterectomy versus simple hysterectomy: aHR=0.82, 95% CI=0.52–1.28, *P*=0.37) and the risk of all-cause death (laparoscopy versus laparotomy: aHR=0.90, 95% CI=0.65–1.26, *P*=0.56. radical hysterectomy versus simple hysterectomy: aHR=0.83, 95% CI=0.58–1.18, *P*=0.29).

For patients of stage II endometrial cancer, using the multivariate analyses, we also found that older than 60 years (aHR=1.75), BMI greater than 24 kg/m^2^ (aHR=1.69), poorly differentiation of tumor (aHR=2.14), and LVSI (aHR=10.90) could increase the risk of cancer recurrence and death caused by cancer of the endometrium. The variables mentioned above also increase the risk of all-cause mortality in patients with stage II endometrial cancer.

The results of the Cox regression analyses are presented in **Table 4**.

**Table 4:** Multivariate analyses of survival for stage II endometrial cancer.

	DFS^a^			OS^b^		
	aHR^c^	95% CI^d^	*P*	aHR	95% CI	*P*
Age at diagnosis						
< 60 years	1			1		
≥ 60 years	1.75	1.16-2.63	0.01	2.26	1.62-3.15	0.00
BMI^e^ at diagnosis						
< 24 kg/m^2^	1			1		
≥ 24 kg/m^2^	1.69	1.08-2.71	0.03	1.75	1.33-2.58	0.01
Grade						
I/II	1			1		
III or undifferentiated	2.14	1.38-3.32	0.00	1.78	1.24-2.56	0.00
Tumor size						
< 4 cm	1			1		
≥ 4 cm	1.46	0.97-2.19	0.07	1.19	0.86-1.66	0.29
LVSI^f^						
No	1			1		
Yes	10.90	6.77-17.54	0.00	10.13	6.77-15.15	0.00
Peritoneal cytology						
Negative	1			1		
Positive	1.34	0.58-1.73	0.56	1.29	0.37-1.72	0.61
Surgical approach						
Laparotomy	1			1		
Laparoscopy	1.04	0.68-1.60	0.85	0.90	0.65-1.26	0.56
Type of hysterectomy						
Simple hysterectomy	1			1		
Radical hysterectomy	0.82	0.52-1.28	0.37	0.83	0.58-1.18	0.29
Adjuvant therapy						
No	1			1		
CT^g^ or RT^h^	1.01	0.63-1.61	0.98	1.09	0.75-1.58	0.65
CT plus RT	0.64	0.32-1.29	0.21	0.59	0.27-1.26	0.33

^a^Disease-free survival.

^b^Overall survival.

^c^Adjusted Hazard Ratio.

^d^Confidence interval.

^e^Body mass index.

^f^Lymphovascular space invasion.

^g^Chemotherapy.

^h^Radiotherapy.

## Discussion

By reviewing and analyzing the data of 684 cases from two Chinese high-volume hospitals, our study shows that for stage II cancer of the endometrium, patients who got radical hysterectomy experienced similar oncological survival outcomes when compared with those who were managed by simple hysterectomy. Our study also shows that the approach of surgical staging does not affect the risk of disease recurrence and death of women with stage II cancer of the endometrium.

The necessity of radical hysterectomy for women with FIGO 2009 stage II cancer of the endometrium has been controversial. Part of the published study has shown that the type of hysterectomy (radical hysterectomy versus simple hysterectomy) is not associated with the risk of disease recurrence and all-cause death in women with stage II cancer of the endometrium (14–16, 26–28). However, there are also different viewpoints. A retrospective cohort study including 577 cases even showed that radical hysterectomy was associated with the deterioration of survival outcomes of patients with high-risk stage II endometrial cancer (17). By reviewing the data from the SEER database, the authors found that both the 5-year OS (62.31% vs. 78.48%, *p* < 0.001) and 5-year cancer-specific survival (74.60 vs. 85.38%, *p* = 0.01) were shorter in radical hysterectomy group than in simple hysterectomy followed by adjuvant radiotherapy (17). Including 10 retrospective cohort studies enrolling 2866 patients, a meta-analysis published in 2019 showed that women who underwent radical hysterectomy did not experience a significant survival benefit for either OS (pooled HR 0.92; 95% CI 0.72–1.16; P = 0.484) or DFS (pooled HR 0.92; 95% CI 0.72–1.16; P = 0.484) (29). The result remained valid after it was balanced with possible impact from other variables that can affect the survival outcomes (29). The findings of our study are consistent with the majority of the studies regarding the topic of endometrial cancer (14–16, 26, 27, 29). We think the main reason why the type of hysterectomy does not affect the survival outcomes of stage II endometrial cancer is that the main invasion path of endometrial cancer and cervical cancer is different (1–3, 30). Although cancer invades the cervix, the risk of involvement of paracervical tissues in stage II endometrial cancer is less than that of cervical cancer. However, all the mentioned studies were retrospective, further investigation is warranted.

For apparent early-stage endometrial cancer, pooled analyses of high-quality clinical research comparing minimally invasive with open surgeries showed that minimally invasive surgery is oncologically safe (31–33). Our study also found that minimally invasive hysterectomy is feasible and safe for patients with stage II endometrial cancer. In 2018, several authors questioned the safety of minimally invasive surgery for women with cervical cancer through their studies (8, 9). They thought the following factors might deteriorate the survival of patients with cervical cancer who underwent minimally invasive surgery: the routine use of a uterine manipulator and the insufflation gas when performing laparoscopy (34, 35). However, the findings of their studies are not valid among apparent early-stage endometrial cancer. Many high-quality studies have confirmed the safety and feasibility of minimally invasive hysterectomy for early-stage cancer of the endometrium (36, 37). The underlying reasons why minimally invasive hysterectomy is safe for early-stage cancer of the endometrium but not safe for early-stage cervical cancer are unclear, our study was not designed to answer this question, so further studies are in need. At the same time, we suspect that the underlying mechanism may be related to the different invasive routes of endometrial cancer and cervical cancer.

Based on two high-volume Chinese centers, our study included 684 cases, this is a relatively large sample. Also, the median duration of follow-up for the study cohort was 77.0 months (range: 0–142), this was a long-term follow-up. however, there are several limitations in our study. First, like most retrospective studies, our study inevitably has some bias, including but not limited to selection bias and recall bias. Second, due to the limited resources, some important and sufficient clinical variables were missing from the extracted case information, such as the specific protocol of postoperative adjuvant therapy and the specific number of cycles of postoperative radiotherapy and/or chemotherapy. Third, nodal status is one of the most important prognostic factors for patients with endometrial cancer and the role of retroperitoneal staging is controversial. In particular, in recent years, more and more studies have been conducted on the relationship between sentinel lymph node detection and prognosis of endometrial cancer (38–40). However, since this technology has not been widely implemented in the participating institutions, we lack relevant raw data, so we do not discuss it further. Fifth, considering the significance of the new FIGO 2023 staging and molecular classification, it would be valuable to incorporate this aspect when evaluating OS and DFS in endometrial cancer. However, the patients in our study were treated between 2011 and 2018 when molecular analysis was not widely available. The last, because of the heterogeneity that exists between different institutions and surgeons, the term ‘radical hysterectomy’ refers to different types of radical hysterectomy. This may deteriorate the integrality and credibility of the analysis in our study.

## Conclusion

For long-term oncological survival, radical hysterectomy is not superior to total hysterectomy in stage II endometrial cancer. Also, for stage II cancer of the endometrium, laparoscopic hysterectomy is as oncologically safe as open hysterectomy.

## Data availability statement

The raw data supporting the conclusions of this article will be made available by the authors, without undue reservation.

## Ethics statement

The studies involving humans were approved by Chengdu Fifth People’s Hospital, West China Second University Hospital. The studies were conducted in accordance with the local legislation and institutional requirements. Written informed consent for participation was not required from the participants or the participants’ legal guardians/next of kin in accordance with the national legislation and institutional requirements.

## Author contributions

XS: Conceptualization, Data curation, Formal analysis, Funding acquisition, Investigation, Methodology, Project administration, Resources, Software, Supervision, Validation, Visualization, Writing – original draft, Writing – review & editing. DX: Conceptualization, Data curation, Formal Analysis, Funding acquisition, Investigation, Methodology, Project administration, Resources, Software, Supervision, Validation, Visualization, Writing – review & editing. BH: Conceptualization, Data curation, Formal analysis, Funding acquisition, Investigation, Methodology, Project administration, Resources, Software, Supervision, Validation, Visualization, Writing – review & editing. YD: Conceptualization, Data curation, Formal analysis, Funding acquisition, Investigation, Methodology, Project administration, Resources, Software, Supervision, Validation, Visualization, Writing – review & editing.

## References

[1] Crosbie EJ Kitson SJ McAlpine JN Mukhopadhyay A Powell ME Singh N. Endometrial cancer. Lancet (London England). (2022) 399:1412–28. doi: 10.1016/S0140-6736(22)00323-3 35397864

[2] Lu KH Broaddus RR. Endometrial cancer. New Engl J Med. (2020) 383:2053–64. doi: 10.1056/NEJMra1514010 33207095

[3] Makker V MacKay H Ray-Coquard I Levine DA Westin SN Aoki D. Endometrial cancer. Nat Rev Dis Primers. (2021) 7:88. doi: 10.1038/s41572-021-00324-8 34887451 PMC9421940

[4] Gu B Shang X Yan M Li X Wang W Wang Q. Variations in incidence and mortality rates of endometrial cancer at the global, regional, and national levels, 1990–2019. Gynecologic Oncol. (2021) 161:573–80. doi: 10.1016/j.ygyno.2021.01.036 33551200

[5] Morice P Leary A Creutzberg C Abu-Rustum N Darai E. Endometrial cancer. Lancet (London England). (2016) 387:1094–108. doi: 10.1016/S0140-6736(15)00130-0 26354523

[6] Concin N Matias-Guiu X Vergote I Cibula D Mirza MR Marnitz S. ESGO/ESTRO/ESP guidelines for the management of patients with endometrial carcinoma. Int J gynecological cancer: Off J Int Gynecological Cancer Soc. (2021) 31:12–39. doi: 10.1136/ijgc-2020-002230 33397713

[7] Aune D Navarro Rosenblatt DA Chan DS Vingeliene S Abar L Vieira AR. Anthropometric factors and endometrial cancer risk: a systematic review and dose-response meta-analysis of prospective studies. Ann oncology: Off J Eur Soc Med Oncol. (2015) 26:1635–48. doi: 10.1093/annonc/mdv142 25791635

[8] Melamed A Margul DJ Chen L Keating NL Del Carmen MG Yang J. Survival after minimally invasive radical hysterectomy for early-stage cervical cancer. New Engl J Med. (2018) 379:1905–14. doi: 10.1056/NEJMoa1804923 PMC6464372 30379613

[9] Ramirez PT Frumovitz M Pareja R Lopez A Vieira M Ribeiro R. Minimally invasive versus abdominal radical hysterectomy for cervical cancer. New Engl J Med. (2018) 379:1895–904. doi: 10.1056/NEJMoa1806395 30380365

[10] Nitecki R Ramirez PT Frumovitz M Krause KJ Tergas AI Wright JD. Survival after minimally invasive vs open radical hysterectomy for early-stage cervical cancer: A systematic review and meta-analysis. JAMA Oncol. (2020) 6:1019–27. doi: 10.1001/jamaoncol.2020.1694 PMC7290695 32525511

[11] Obermair A Asher R Pareja R Frumovitz M Lopez A Moretti-Marques R. Incidence of adverse events in minimally invasive vs open radical hysterectomy in early cervical cancer: results of a randomized controlled trial. Am J Obstetrics Gynecology. (2020) 222:249.e241–249.e210. doi: 10.1016/j.ajog.2019.09.036 PMC7181470 31586602

[12] Touhami O Plante M. Minimally invasive surgery for cervical cancer in light of the LACC trial: what have we learned? Curr Oncol (Toronto Ont). (2022) 29:1093–106. doi: 10.3390/curroncol29020093 PMC8871281 35200592

[13] Vergote I Magrina JF Zanagnolo V Magtibay PM Butler K Gil-Moreno A. The LACC trial and minimally invasive surgery in cervical cancer. J Minimally Invasive Gynecology. (2020) 27:462–3. doi: 10.1016/j.jmig.2019.09.767 31520725

[14] Barquet-Muñoz SA Cantú-de-León D Bandala-Jacques A González-Enciso A Isla-Ortiz D Prada D. What is the impact of radical hysterectomy on endometrial cancer with cervical involvement? World J Surg Oncol. (2020) 18:101. doi: 10.1186/s12957-020-01876-x 32438919 PMC7243320

[15] Lennox GK Clark M Zigras T Rouzbahman M Han G Bernardini MQ. Does radical hysterectomy for clinically apparent stage II endometrial cancer affect risk of local recurrence? J obstetrics gynaecology Canada: JOGC = J d'obstetrique gynecologie du Canada: JOGC. (2021) 43:564–70. doi: 10.1016/j.jogc.2020.12.017 33412305

[16] Nasioudis D Sakamuri S Ko EM Haggerty AF Giuntoli RL 2nd Burger RA. Radical hysterectomy is not associated with a survival benefit for patients with stage II endometrial carcinoma. Gynecologic Oncol. (2020) 157:335–9. doi: 10.1016/j.ygyno.2020.02.003 32089334

[17] Wang M Ran R Wu Y. Radical hysterectomy versus simple hysterectomy and brachytherapy for stage II endometrial cancer. J Obstetrics Gynaecology Res. (2021) 47:3943–50. doi: 10.1111/jog.14988 34409683

[18] World Medical Association Declaration of Helsinki: ethical principles for medical research involving human subjects. JAMA. (2013) 310:2191–4. doi: 10.1001/jama.2013.281053 24141714

[19] Querleu D Cibula D Abu-Rustum NR. 2017 Update on the querleu-Morrow classification of radical hysterectomy. Ann Surg Oncol. (2017) 24:3406–12. doi: 10.1245/s10434-017-6031-z PMC6093205 28785898

[20] Karran JC Moodie EE Wallace MP. Statistical method use in public health research. Scandinavian J Public Health. (2015) 43:776–82. doi: 10.1177/1403494815592735 26163023

[21] Mishra P Pandey CM Singh U Keshri A Sabaretnam M. Selection of appropriate statistical methods for data analysis. Ann Cardiac Anaesthesia. (2019) 22:297–301. doi: 10.4103/aca.ACA_248_18 PMC6639881 31274493

[22] Bull K Spiegelhalter DJ. Survival analysis in observational studies. Stat Med. (1997) 16:1041–74. doi: 10.1002/(ISSN)1097-0258 9160498

[23] Sebastião YV St Peter SD. An overview of commonly used statistical methods in clinical research. Semin Pediatr Surg. (2018) 27:367–74. doi: 10.1053/j.sempedsurg.2018.10.008 30473041

[24] Stolberg HO Norman G Trop I. Survival analysis. AJR Am J roentgenology. (2005) 185:19–22. doi: 10.2214/ajr.185.1.01850019 15972392

[25] Koletsi D Pandis N. Survival analysis, part 3: Cox regression. Am J orthodontics dentofacial orthopedics: Off Publ Am Assoc Orthodontists its constituent societies Am Board Orthodontics. (2017) 152:722–3. doi: 10.1016/j.ajodo.2017.07.009 29103451

[26] Jiang Y Jia N Zhu M He Y Che X Lv T. Comparison of survival and perioperative outcomes following simple and radical hysterectomy for stage II endometrial cancer: a single-institution, retrospective, matched-pair analysis. J Int Med Res. (2019) 47:4469–81. doi: 10.1177/0300060519863190 PMC6753566 31357882

[27] Hasegawa T Furugori M Kubota K Asai-Sato M Yashiro-Kawano A Kato H. Does the extension of the type of hysterectomy contribute to the local control of endometrial cancer? Int J Clin Oncol. (2019) 24:1129–36. doi: 10.1007/s10147-019-01458-2 PMC6687671 31069549

[28] Vetter MH Bixel K Felix AS. Management of stage II endometrial cancer and subsequent oncologic outcomes: a National Cancer Database study. J Gynecologic Oncol. (2020) 31:e84. doi: 10.3802/jgo.2020.31.e84 PMC7593216 33078593

[29] Liu T Tu H Li Y Liu Z Liu G Gu H. Impact of radical hysterectomy versus simple hysterectomy on survival of patients with stage 2 endometrial cancer: A meta-analysis. Ann Surg Oncol. (2019) 26:2933–42. doi: 10.1245/s10434-019-07472-y 31147990

[30] Johnson CA James D Marzan A Armaos M. Cervical cancer: an overview of pathophysiology and management. Semin Oncol Nurs. (2019) 35:166–74. doi: 10.1016/j.soncn.2019.02.003 30878194

[31] Ghazali W Jamil SA Sharin IA. Laparoscopic versus laparotomy: staging surgery for endometrial cancer - Malaysia's early experience. Gynecology minimally invasive Ther. (2019) 8:25–9. doi: 10.4103/GMIT.GMIT_25_18 PMC6367911 30783585

[32] Vardar MA Gulec UK Guzel AB Gumurdulu D Khatib G Seydaoglu G. Laparoscopic surgery for low, intermediate and high-risk endometrial cancer. J Gynecologic Oncol. (2019) 30:e24. doi: 10.3802/jgo.2019.30.e24 PMC6393633 30740955

[33] Uccella S Bonzini M Palomba S Fanfani F Malzoni M Ceccaroni M. Laparoscopic vs. open treatment of endometrial cancer in the elderly and very elderly: An age-stratified multicenter study on 1606 women. Gynecologic Oncol. (2016) 141:211–7. doi: 10.1016/j.ygyno.2016.02.029 26920107

[34] Scutiero G Vizzielli G Taliento C Bernardi G Martinello R Cianci S. Influence of uterine manipulator on oncological outcome in minimally invasive surgery of endometrial cancer: A systematic review and meta-analysis. Eur J Surg oncology: J Eur Soc Surg Oncol Br Assoc Surg Oncol. (2022) 48:2112–8. doi: 10.1016/j.ejso.2022.05.034 35725683

[35] Zorzato PC Uccella S Biancotto G Bosco M Festi A Franchi M. Intrauterine manipulator during hysterectomy for endometrial cancer: a systematic review and meta-analysis of oncologic outcomes. Am J Obstetrics Gynecology. (2024) 230:185–198.e184. doi: 10.1016/j.ajog.2023.09.004 37704174

[36] Janda M Gebski V Davies LC Forder P Brand A Hogg R. Effect of total laparoscopic hysterectomy vs total abdominal hysterectomy on disease-free survival among women with stage I endometrial cancer: A randomized clinical trial. JAMA. (2017) 317:1224–33. doi: 10.1001/jama.2017.2068 28350928

[37] Walker JL Piedmonte MR Spirtos NM Eisenkop SM Schlaerth JB Mannel RS. Recurrence and survival after random assignment to laparoscopy versus laparotomy for comprehensive surgical staging of uterine cancer: Gynecologic Oncology Group LAP2 Study. J Clin oncology: Off J Am Soc Clin Oncol. (2012) 30:695–700. doi: 10.1200/JCO.2011.38.8645 PMC3295548 22291074

[38] Cuccu I Raspagliesi F Malzoni M Vizza E Papadia A Di Donato V. Sentinel node mapping in high-intermediate and high-risk endometrial cancer: Analysis of 5-year oncologic outcomes. Eur J Surg oncology: J Eur Soc Surg Oncol Br Assoc Surg Oncol. (2024) 50:108018. doi: 10.1016/j.ejso.2024.108018 38428106

[39] Bogani G Giannini A Vizza E Di Donato V Raspagliesi F. Sentinel node mapping in endometrial cancer. J Gynecologic Oncol. (2024) 35:e29. doi: 10.3802/jgo.2024.35.e29 PMC10792208 37973163

[40] Restaino S Paglietti C Arcieri M Biasioli A Della Martina M Mariuzzi L. Management of patients diagnosed with endometrial cancer: comparison of guidelines. Cancers. (2023) 15. doi: 10.3390/cancers15041091 PMC9954548 36831434

